# Evaluation of Data Errors and Monitoring Activities in a Trial in Japan Using a Risk-Based Approach Including Central Monitoring and Site Risk Assessment

**DOI:** 10.1007/s43441-021-00286-9

**Published:** 2021-04-19

**Authors:** Hidenobu Kondo, Tomoaki Kamiyoshihara, Kenji Fujisawa, Toshiaki Nojima, Ryohei Tanigawa, Hisataka Fujiwara, Hideki Suganami, Yukikazu Hayashi, Takuhiro Yamaguchi

**Affiliations:** 1 Clinical Operation Steering Department, A2 Healthcare Corporation, Tokyo, Japan; 2 Division of Biostatistics, Tohoku University Graduate School of Medicine, Sendai, Japan; 3 Clinical Development Department I, A2 Healthcare Corporation, Tokyo, Japan; 4 Clinical Data Science Department, Pharmaceutical Division Kowa Company, Ltd., Tokyo, Japan; 5 Clinical Development Department, Pharmaceutical Division Kowa Company, Ltd., Tokyo, Japan; 6 Datascience Division, A2 Healthcare Corporation, Tokyo, Japan; 7 Department of Clinical Trial Data Management, Graduate School of Medicine, The University of Tokyo, Tokyo, Japan

**Keywords:** Risk-based monitoring, RBM, Central monitoring, Site risk assessment

## Abstract

**Background:**

Risk-based monitoring (RBM) is a slow uptake in some trial sponsors. There are three main reasons for this. First, there is the fear of making large investments into advanced RBM technology solutions. Second, it is considered that RBM is most suitable for large, complex trials. Third, there is the fear of errors in both critical and non-critical data, appearing as reduced on-site monitoring is being conducted.

**Methods:**

Our RBM team identified, evaluated, and mitigated trial risks, as well as devised a monitoring strategy. The clinical research associate (CRA) assessed the site risks, and the RBM team conducted central monitoring. We compared all data errors and on-site monitoring time between the partial switching sites [sites that had switched to partial source data verification (SDV) and source data review (SDR)] and the 100% SDV and SDR sites (sites that had implemented 100% SDV and SDR).

**Results:**

Partial switching sites did not require any critical data correction and had a smaller number of data corrections through on-site monitoring than the 100% SDV and SDR sites. The RBM strategy reduced the on-site monitoring time by 30%.

**Conclusions:**

The results suggest that RBM can be successfully implemented through the use of site risk assessment and central monitoring with practically no additional investment in technology and still produced similar results in terms of subject safety and data quality, as well as the cost savings that have been reported in global complex studies.

## Introduction

Risk-based monitoring (RBM) was first introduced into studies in 2011–2012. By the time ICH E6(R2) was issued, RBM was already a well-known concept. The FDA, EMA, and PMDA had included it in their regulations for clinical trials, making it mandatory in their jurisdictions. The upcoming ICH E6(R3) in 2021 is expected to strengthen it even further. However, there has been rather slow uptake by some trial sponsors all over the world. There are three main reasons for this. First, there is a fear of making large investments in advanced RBM technology solutions. Second, it is thought that RBM is only suitable for large complex trials. Third, there is a fear of erroneous data, in both critical and non-critical data when the on-site monitoring is reduced.

According to ICH E6(R2), sponsors should develop systems to manage quality throughout all stages of the trial process, and quality management systems should use a risk-based approach (RBA). An RBA process is commonly known as RBM [1]; this approach has been taken in many contexts worldwide, and many pharmaceutical companies are implementing targeted monitoring [partial source data verification (SDV) and source data review (SDR)] for critical data and/or processes, focusing on the need to protect human subjects and ensure the reliability of trial results. However, reports of trials that have evaluated RBM methods are very few, including the partial SDV and/or SDR, central monitoring, and site risk assessments.

Sheetz et al*.* showed that a median of 1.1% of the total eCRF datasets in 1168 phase I–IV biopharmaceutical studies performed with 53 sponsors was corrected by SDV [2]. This analysis was retrospective, and the corrections, driven by SDV and SDR, both critical and non-critical, were not differentiated. Andersen et al*.* reported that in three randomized, double-blind, placebo-controlled, multicenter phase III trials, 100% SDV yielded an error rate of 0.27%, and the error rate for partial SDV was 0.53% [3]. That study did not distinguish between SDV and SDR. In the ADAMON cluster-randomized study, 213 sites were randomized between extensive on-site monitoring and risk-adapted monitoring. Here, extensive on-site and risk-adapted monitoring included SDV, which checked the existence of trial subjects and informed consent documentation. It investigated whether risk-adapted monitoring strategies were as effective as extensive on-site monitoring strategies in preventing major or critical violations of GCP objectives, as ascertained by independent audits at the end of the trial. The study showed that risk-adapted monitoring requires less than 50% of the resources needed for extensive on-site monitoring, and the rate of GCP violations for risk-adapted monitoring was non-inferior to those found in extensive monitoring [4]. In the Optimon trial, the researchers randomized sites into either a 100% on-site strategy or a risk-adapted strategy. The main outcome of the Optimon trial was a proportion of participant charts without non-conformities for consent signature, SAE notifications, main eligibility criteria, and main study outcome. In the study, the non-inferiority of the risk-adapted strategy was not demonstrated [5]. Higa et al. showed that RBM supports the reliability of the trial results and promotes efficient monitoring based on the outcome of a PMDA inspection [6].

However, no RBM studies have been performed in relation to all errors and deviations of eCRF data and source data as detected by central monitoring, off-site monitoring, and on-site monitoring, including SDV and SDR. No observations have yet been conducted on the combination of central monitoring and site risk assessment as seen in previous studies.

We implemented RBM in our trial with the three-fold objective of investigating the following:

1. Whether a simple RBM without advanced technology will produce satisfactory results in terms of managing data and safety risks.
2. Whether an RBM provides benefits in a low-risk trial with minimal complexity.
3. Whether an RBM can ensure quality regarding all errors in eCRF and source data, minimize protocol deviations, and optimize the monitoring resource utilization.

We included non-critical data errors to simulate traditional monitoring strategies with 100% SDV and SDR. We compared errors and on-site monitoring time between sites that switched to partial SDV and SDR and sites with 100% SDV and SDR.

## Methods

### Trial Information

Our study was an open-label, multicenter trial performed to evaluate 52-week long-term safety, tolerability, and efficacy of tofogliflozin in conjunction with glucagon-like peptide-1 (GLP-1) analogue treatment in type 2 diabetes mellitus. The 67 participants at 11 sites in Japan were randomized. The primary endpoint for this trial was change from baseline in HbA1c. Trial data were collected using electronic data capture (EDC). Data freezing was conducted at each site.

The trial sponsors were Kowa Company, Ltd. and Sanofi K.K. The trial was managed and conducted by A2 Healthcare Corporation, a Japanese contract research organization. The members of Kowa Company, Ltd. and A2 Healthcare Corporation discussed and determined the RBM strategy. This trial was registered at ClinicalTrials.gov with number NCT02537834.

### Risk Identification, Risk Assessment, and Risk Mitigation Plan

We identified and assessed trial risk with the TransCelerate Risk Assessment and Categorization Tool (RACT). We formulated a risk mitigation plan for the identified risks. This was performed by the monitoring leader and the RBM team members, including central monitors, data managers (DMs), and statisticians. The risk mitigation plan was adapted to accommodate manuals and/or training for site staff, patient diaries, and discussion of site processes with site staff. The discussions were conducted at all sites, and the results of the discussion were recorded in the process-building sheet, which included an optimized clinical process designed to prevent protocol deviations.

### Monitoring Plan

We located sites that switched to partial SDV and SDR. These were referred to as partial switching sites, using information related to the previous involvement of the trial sponsor (Kowa Company, Ltd.) and the results of the site risk assessments performed by the CRA. The assessed sites were those where the CRA had implemented monitoring in other trials. The CRA determined whether issues had arisen that could affect subject safety or the quality of the trial data, including non-reporting or late reporting of SAEs, late EDC entry, or inadequately trained site staff. Using information on the previous involvement of the sponsor and the site risk assessments, all sites for which information was already present were termed partial switching site.

At the beginning of the trial, the CRA discussed the site processes with the site staff at all sites and completed the process-building sheet. At the partial switching sites, where the process-building sheets were completed, the CRA assessed the site risks and switched to partial SDV and SDR at the low-risk sites, using a Monitor-driven Risk Assessment Categorization Tool (MRACT). Until that point, the CRA had performed 100% SDV and SDR at these sites. MRACT included the items necessary to identify the issues and risks that would be difficult to detect with data-driven risk indicators, such as the quality of the source documentation. At the 100% SDV and SDR sites, which referred to all sites that were not partial switching sites and for which there was no information before starting the trial, the CRA also carried out an assessment using MRACT to evaluate the site risks without switching. The implementation details for on-site monitoring, off-site monitoring, and central monitoring activities for this trial are presented in Table 1. The data volume and range differ for the different monitoring methods. SDV was implemented after the site staff entered and froze the data.

**Table 1:** Summary of On-Site, Off-Site, and Central Monitoring Activities.

*On-site monitoring at “100% SDV and SDR sites”* Each site was required to implement the following activities by CRA about once a month: • All data verified against source documentation at the site • All source documentation reviewed at the site • Regulatory file review (including informed consent) • Drug management and accountability review *On-site monitoring at “partial switching sites”* After switching to a “partial switching site,” each site was implemented the following activities every 3 months by CRA: • Only for the first randomized subject (if the first subject dropped out, discontinued, or completed, the monitored subject was switched to the last randomized subject at that time) was 100% SDV and SDR implemented. In other subjects, only SDR for consent and eligibility and SDV for Interactive Web Response System (IWRS) data were performed. In cases where SAEs, medically important events, discontinuations, or major deviations occurred, the relevant source documents were implemented for SDR • Regulatory file review (including informed consent) • Drug management and accountability review After the last patient last visit, the CRA applied 100% SDV and SDR to the data and processes which were not monitored during the trial *Off-site monitoring* All sites underwent weekly data review, query creating at EDC, query closing at EDC and query by telephone or email. The CRA performed SDV and/or SDR after the DM had reviewed the data. The order of data review by DM and SDV and/or SDR by CRA was not specified for the following: • SDR of treatment compliance, GLP-1 receptor agonist compliance, adverse events, concomitant medications, SAEs, discontinuations, and items related to major deviations • SDV and SDR for eligibility prior to discussion on the site processes and completion of the process-building sheet *Central monitoring* Central monitoring was performed monthly referring to the results of the pre-specified key risk indicators (KRIs) and MRACT. Assessment of MRACT was carried out by the CRA on a monthly basis. The persons responsible for central monitoring discussed the results of MRACT and central monitoring at a meeting. The RBM team members recommended that the CRAs take preventive actions when necessary. If the site staff encountered the same issues in spite of implementing corrective action, or there were critical protocol deviations or non-reported SAEs, we switched the site from a partial switching site to a 100% SDV and SDR site. A summary of the KRIs is provided below: • Site performance (e.g., data freezing, answering queries and staff changes) • Protocol deviations • Screening failure and subject discontinuation • Study drug compliance • Adverse events, serious adverse events, and adverse events of special interest	

**Figure 1:**
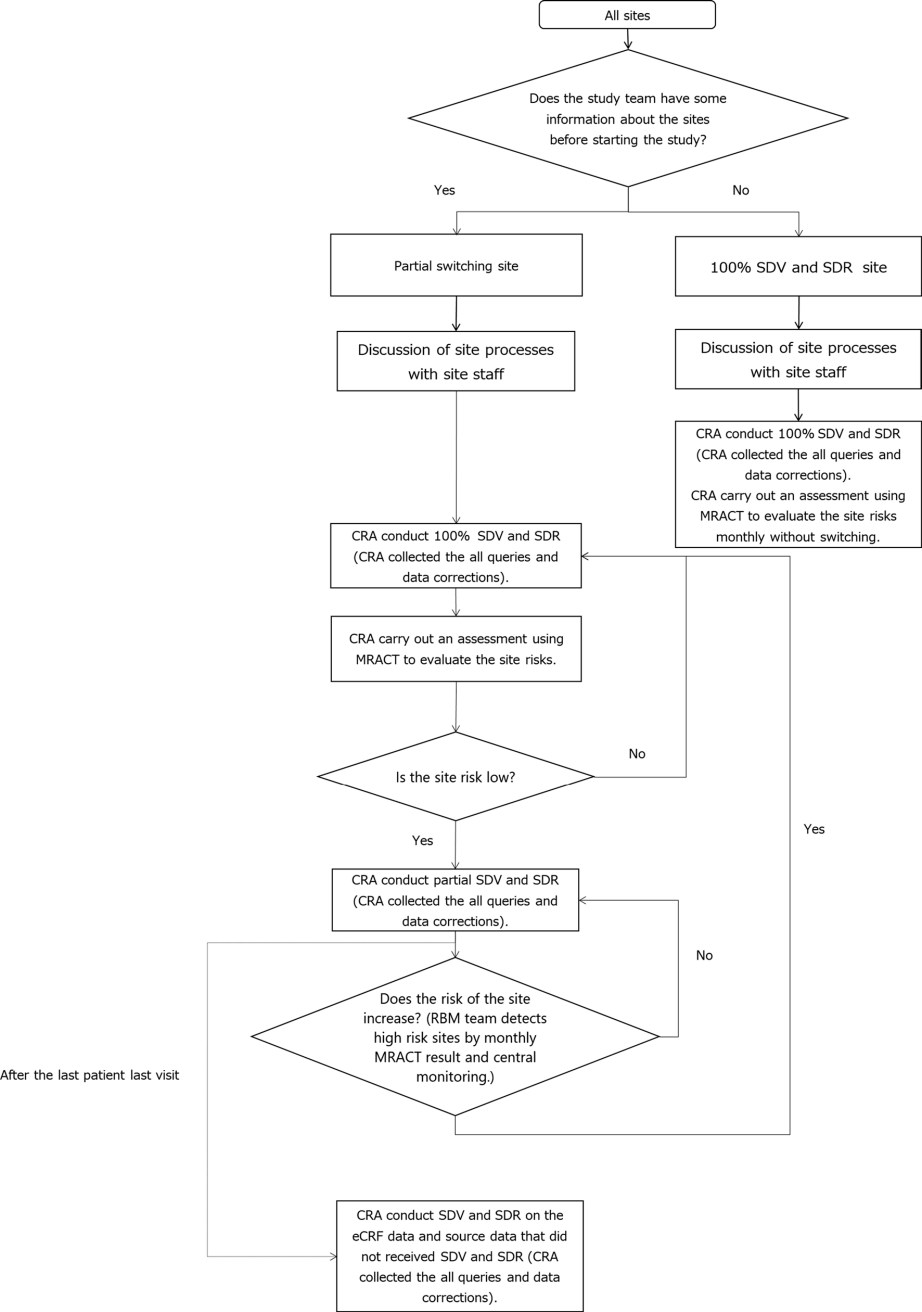
Diagram of monitoring plan.

After the last patient last visit, the CRA conducted SDV and SDR on the data that had not received SDV and SDR at the partial switching sites during the trial implementation phase (Fig. 1).

### Site Risk Assessment

MRACT consists of 17 checklist items, categorized into three groups: critical processes (four items), data quality (three items), and staffing and facilities (ten items). Each checklist item had a risk score from 1 to 3 (1 = low risk, 2 = medium risk, 3 = high risk) in accordance with the risk assessment criteria. The CRA began the assessment of the site using MRACT items following the first on-site monitoring. After the first assessment, the CRA carried out monthly assessments at each site, using MRACT. All of the results of the assessments were reviewed and approved by the CRA leader. The RBM team members reviewed the results and queried the CRA leader where necessary. The implementation of the review and the approval minimized the bias and subjectivity of the site risk assessment. The MRACT was not updated during the trial.

### Error Data Collection

The CRAs collected all queries to the doctor or clinical research coordinator (CRC) in Microsoft Excel software on a data point basis after on-site monitoring and off-site monitoring. The data collected by the CRAs included corrections of the eCRF and the source data. Where data were corrected, the CRA presented the data and the reason for the error in the spreadsheet file. The reasons for the corrections included lack of understanding of the trial protocol, errors in source documents, transcription errors, lack of data entry, and others. To minimize the human error in data entry by the CRAs, the spreadsheet file for the data collection was set to select and input from the drop-down list, and the CRA leader confirmed the data entered by CRA to allow it to be detected when a human error occurred. A team member of central monitoring collected all errors detected by central monitoring in an Excel spreadsheet. The DM extracted the created query data and the data corrected through the DM queries from the EDC audit trail data at the end of the trial. The dates and times of the beginning and the end of the on-site monitoring were collected by the CRAs. The collection of the data continued from the first patient first visit to the last patient last visit for partial switching sites and the 100% SDV and SDR sites. After the last patient last visit, the CRA conducted SDV and SDR on the data that had not received SDV and SDR at the partial switching sites to collect the error data.

### Statistical Analysis for Error Data

The number of corrected eCRF and source data per subject was compared between the 100% SDV and SDR sites and the partial switching sites. The number of corrected eCRF and source data per subject was also compared between monitoring activities. This involved on-site monitoring by the CRA, off-site monitoring by the CRA, off-site monitoring by the DM, central monitoring, and correction by site. The time required for on-site monitoring per completed subject was compared between the 100% SDV and SDR sites and the partial switching sites. The number of corrected eCRF and source data per subject was also compared between the data risk categories, which were determined using the impact of data errors, classified as high, medium, or low. The high-risk category included critical data related to the protection of the human subjects, the reliability of the trial results, and GCP compliance. The high-risk category data were adverse event, primary endpoint, and informed consent. The medium-risk category included secondary endpoint data. The low-risk category included all other data not covered by the high- or medium-risk categories.

To summarize the metrics assessed in this investigation, the following definitions were used:

$$\frac{\begin{gathered} {\text{The number of eCRF and source data corrections per subject at the 1}}00\% {\text{ SDV and}} \hfill \\ {\text{ SDR sites or the partial switching sites }} =\hfill \\ {\text{total eCRF and source data points corrected}} \hfill \\ \end{gathered} }{{\text{total number of subjects}}}$$

$$\frac{\begin{gathered} {\text{The number of eCRF data and source data corrections per subject corrected by the different}}\;{\text{monitoring activities }} = \hfill \\ {\text{total eCRF and source data points corrected by the different monitoring activities}} \hfill \\ \end{gathered} }{{\text{total number of subjects}}}$$

$$\frac{\begin{gathered} {\text{The time for on-site monitoring per completed subject at the 1}}00\% {\text{ SDV and SDR sites or the}}\;{\text{partial switching sites }} = \hfill \\ {\text{total time of on-site monitoring}} \hfill \\ \end{gathered} }{{\text{total number of completed subjects}}}$$

## Results

**Figure 2:**
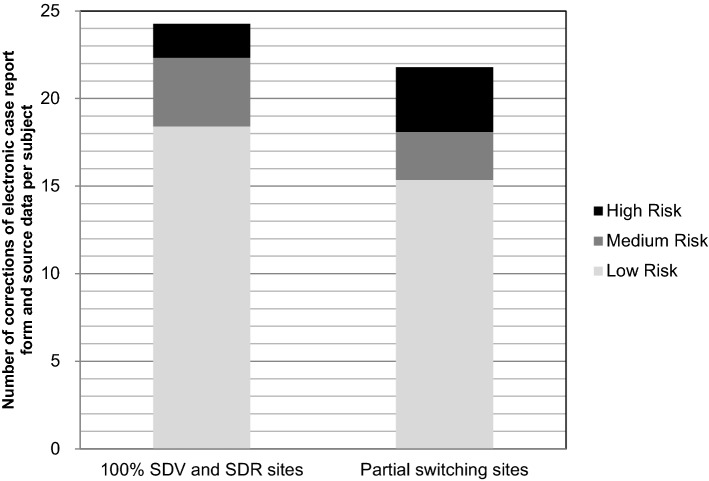
Number of corrections of the electronic case report form and source data for data risk categories at the 100% SDV and SDR sites and the partial switching sites.

**Figure 3:**
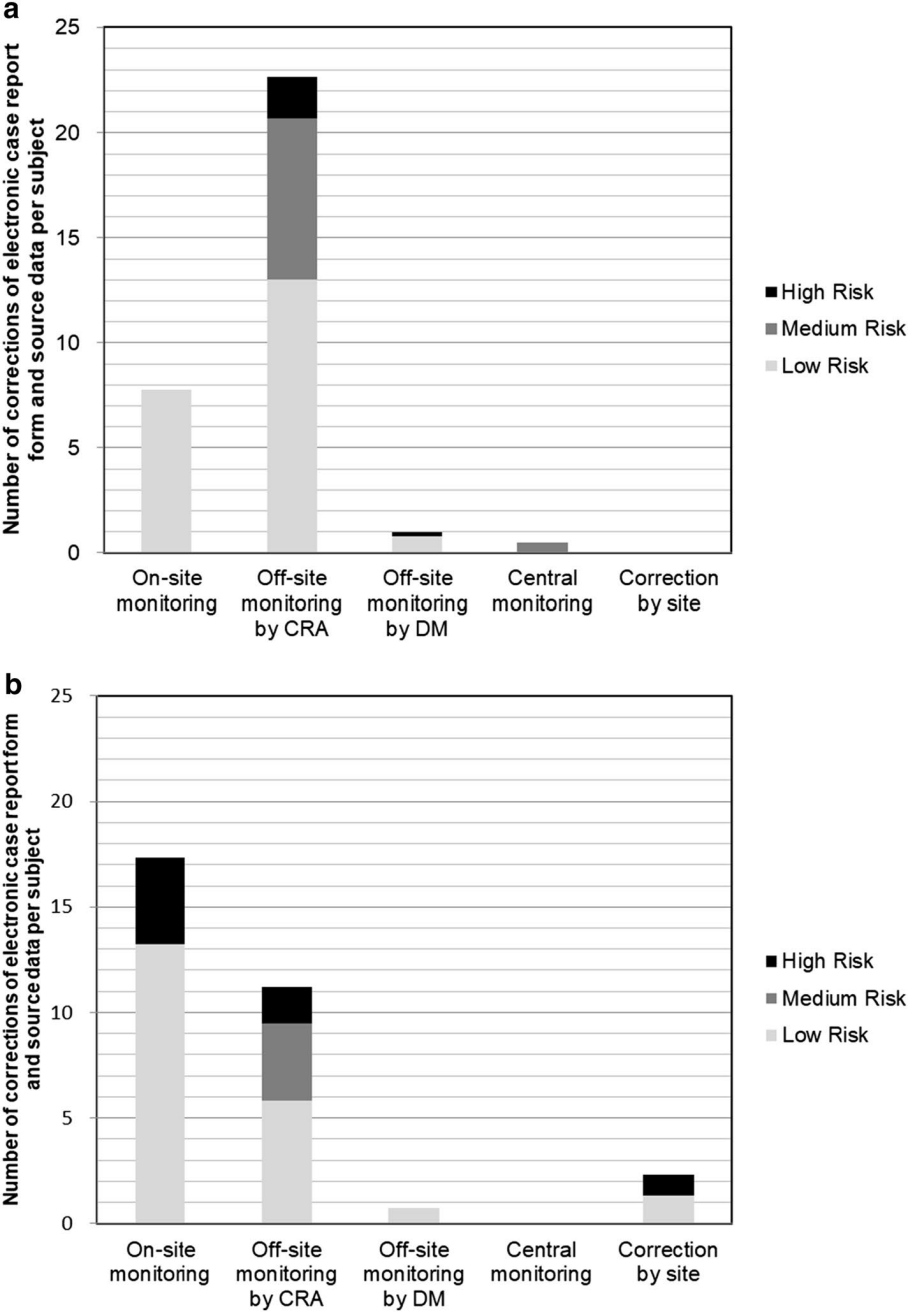
Number of corrections of electronic case report form and source data per monitoring type. **a** Partial switching sites. **b** 100% SDV and SDR sites.

The number of eCRF and source data per subject corrected for the 100% SDV and SDR sites and that for the partial switching sites was nearly equal, at 24.3 and 21.8, respectively (Fig. 2). When SDV and SDR were performed after the completion of the clinical trial at the partial switching sites, no correction of high-risk category data was performed. The number of eCRF and source data corrections per subject by on-site monitoring at the partial switching sites was lower than that at the 100% SDV and SDR sites, and the partial switching sites had only low-risk category correction done by on-site monitoring (Fig. 3). The number of eCRF and source data corrections per subject by off-site monitoring at the partial switching sites was higher than that at the 100% SDV and SDR sites (Fig. 3). Though central monitoring was also set up for the high-risk data, the data correction by central monitoring occurred for medium-risk data only.

**Figure 4:**
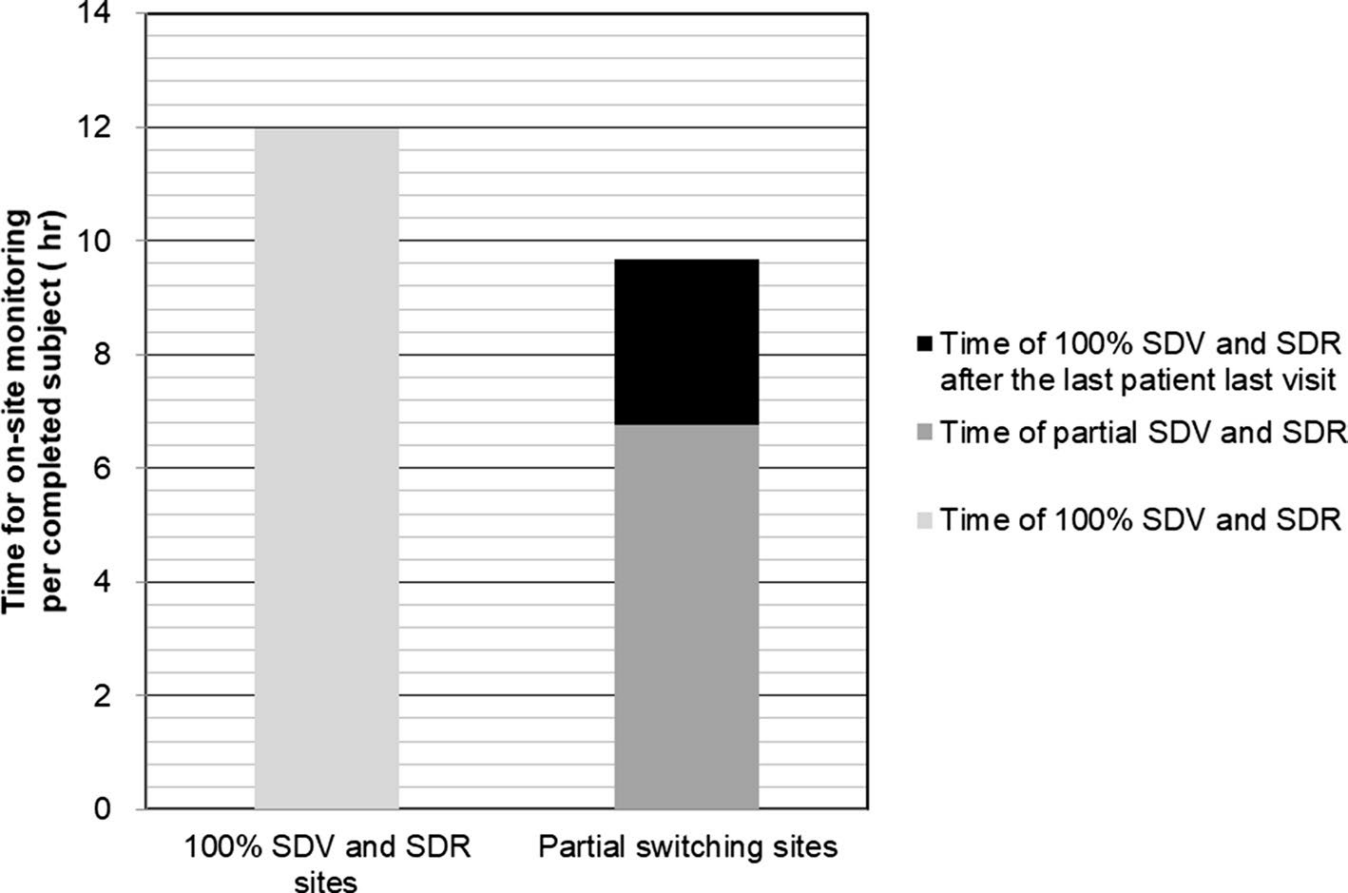
Time required for on-site monitoring at the 100% SDV and SDR sites and the partial switching sites.

Doing on-site monitoring took 11.99 h and 9.67 h per completed subject for the 100% SDV and SDR sites and that spent for the partial switching sites, respectively (Fig. 4). For the partial switching sites, the partial SDV and SDR reduced the on-site monitoring time by 30% (Fig. 4).

**Figure 5:**
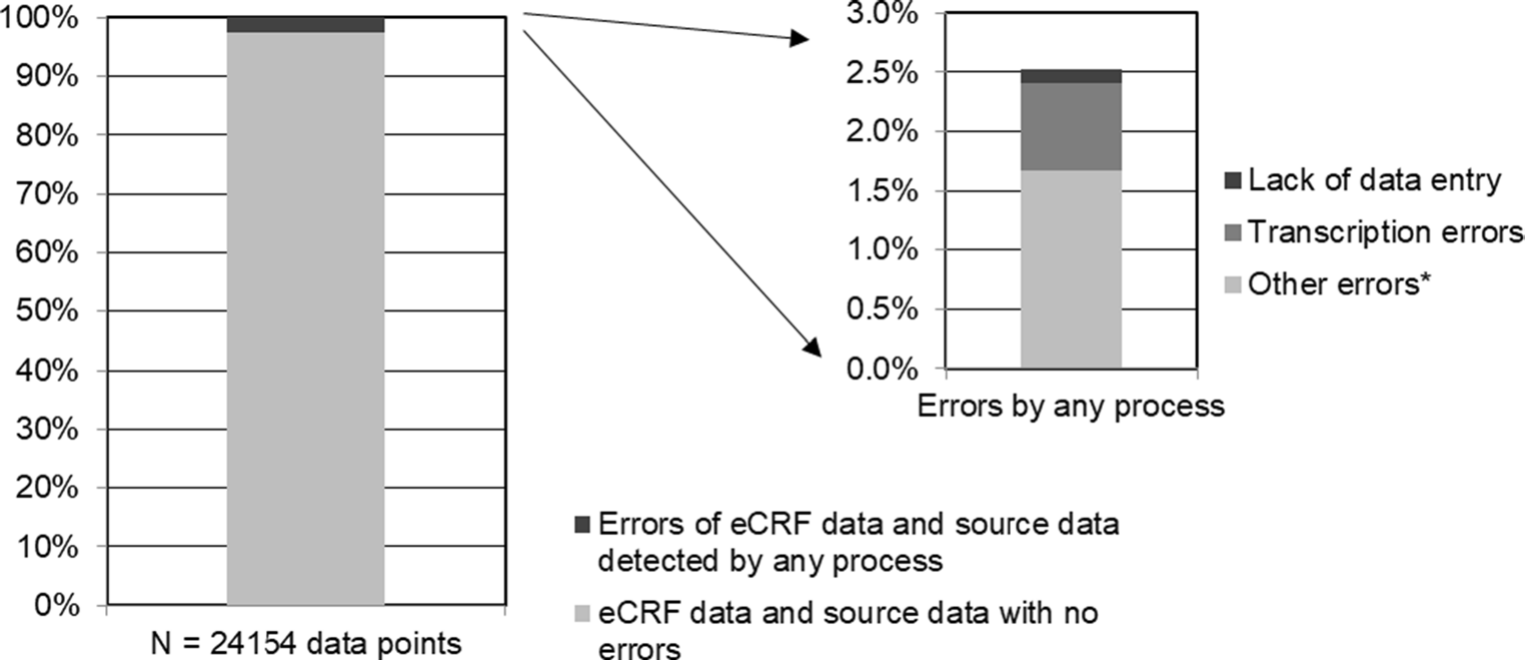
Percentage of errors of eCRF data and source data detected by on-site monitoring. * Reasons for other errors include lack of understanding of the trial protocol and errors in source documents.

The correction rate for on-site monitoring was 2.5% and that for transcription error and lack of data entry was 0.8% in total (Fig. 5).

## Discussion

Between step 2 and step 4 in the evolution of ICH E6(R2), the focus on quality management shifted from ensuring the integrity of the trial data to ensuring the reliability of the trial results. That is, it is important to ascertain what approach might be more effective and efficient. This study investigates whether RBM can ensure the protection of human subjects and the reliability of trial results while optimizing monitoring resources.

The partial switching sites showed no data corrections in high-risk category items. This means that no critical errors occurred at those sites. Our definition of partial switching site was based on our own information, but we did detect one problem site from the MRACT results and central monitoring during the trial implementation phase. After the detection, we switched it from partial SDV and SDR to 100% SDV and SDR. After the switching, we determined the cause of the critical error and implemented corrective action. These results suggest that the strategy of switching the site monitoring based on the MRACT results and central monitoring is an option for RBM strategy that could ensure the reliability of the trial results.

The number of eCRF and source data per subject corrected by off-site monitoring at partial switching sites was higher than that found at 100% SDV and SDR sites. All CRAs were in charge of monitoring the partial switching sites and the 100% SDV and SDR sites. Off-site monitoring activities depended on the CRAs, and all four CRAs stated that the time required and the motivation for off-site monitoring were same for both the partial switching sites and the 100% SDV and SDR sites. These observations suggest that the result cannot be attributed to the differences in the implementation of monitoring among the CRAs. Because the CRAs were not prohibited from conducting on-site monitoring before the weekly off-site monitoring, their on-site monitoring probably detected the errors that the CRAs could detect with weekly off-site monitoring at the 100% SDV and SDR sites.

The number of eCRF and source data corrections per subject with on-site monitoring at the partial switching sites was lower than that at the 100% SDV and SDR sites, and the partial switching sites had only low-risk category correction with on-site monitoring. These results suggest that the strategy of switching site monitoring based on the MRACT results and central monitoring effectively detects data error.

The partial SDV and SDR reduced the on-site monitoring time by 30%. This suggests that the reduction in time spent on on-site monitoring at the partial switching sites could be attributed to the reduction in the frequency of on-site monitoring from once a month to once every three months.

Sheetz et al. showed retrospectively that a median of 1.1% of the total eCRF datasets was corrected by SDV in their data [2], while the corresponding rate in our study was only 0.7%. The correction rate given by Sheetz et al. was calculated only for transcription errors. In our study, the correction rates were calculated prospectively, using the number of data corrections and total number of eCRF data points. We collected the correction reasons that the CRA ascertained and recorded. The correction rate for on-site monitoring was 2.5%, that for transcription error was 0.7%, and that for lack of data entry was 0.1%. We thus ascertained that the contributions were made purely by SDV, and the correction rate was likely lower than that in the previous study. This suggests that SDV should have a greater focus on critical data.

In the ADAMON study, the monitoring intensities were adjusted, but the issues that affected critical data and processes were identified after completion of the trial [3]. On the other hand, we encountered no issues related to either critical data or processes adopted at the partial switching sites in our trial. This suggests that switching to 100% SDV and SDR based on the results of the central monitoring and site risk assessment is effective, as the ADAMON study did not include this switch. Other factors also likely contributed to the different results between the ADAMON study and our study. The first factor of these is the site process discussion at all sites, performed using the process-building sheet. This is a proactive risk mitigation strategy for the sites and probably helped reduce errors at the trial at all sites. The second factor relates to the fact that the complexity of our trial was low.

It should be noted as a limitation of the study that the trial was conducted with a small sample size. Therefore, its results cannot be generalized. It is unclear whether the same results would be obtained if a more complex trial was conducted, as this trial required few complex or uncommon procedures that go beyond the usual standard of care.

We obtained the partial switching sites using prior information. Although this introduces bias, the following two points probably ensured the comparability among the 100% SDV and SDR sites and the partial switching sites.

First, we did not assess or select sites for which no information was available. Accordingly, the 100% SDV and SDR sites probably included low-risk sites, which we could probably defined as partial switching sites.

Second, the information that we used to assess and select of the partial switching sites was probably insufficient. This is because the partial switching sites included a site that was switched from the partial switching sites to the 100% SDV and SDR sites, following the MRACT results and central monitoring. Furthermore, the CRAs held discussions with the site staff at all sites for risk mitigation, assessed all sites using MRACT, and verified that all sites were low risk at the beginning of the trial.

In our study, we suggested that the implementation of RBM can efficiently ensure the quality of trials. Switching strategies are commonly practiced in the industry. However, to our knowledge, this is the first prospective study to demonstrate the effects of RBM, evaluated using all data corrections, adopting a strategy wherein site monitoring can be switched based on the results of site risk assessment and central monitoring.

## Conclusion

Our study shows that RBM can be successfully implemented with site risk assessment and central monitoring with practically no investment in technology and can still produce similar results in terms of subject safety and data quality, along with showing the savings that have been reported in global complex studies. The time saved by the CRAs can be used to perform additional studies or engage in other activities. We believe that this study will provide a convincing support for the wider spread of RBM studies.
